# Real-world disproportionality analysis of sleep disturbances associated with antiseizure medications in epilepsy: a pharmacovigilance study

**DOI:** 10.3389/fphar.2025.1740747

**Published:** 2025-12-12

**Authors:** Chen Gou, Mengshi Yang, Qingqing Zhao, Yanbing Han

**Affiliations:** 1 First Department of Neurology, First Affiliated Hospital, Kunming Medical University, Kunming, Yunnan, China; 2 Yunnan Clinical Center for Neurological and Cardiovascular Diseases, Kunming, Yunnan, China

**Keywords:** antiseizure medications, disproportionality analysis, epilepsy, FAERS, sleep disturbances

## Abstract

**Objective:**

Real-world evidence characterizing the safety profiles of antiseizure medications (ASMs) concerning sleep disturbances remains limited. This study aimed to systematically evaluate the reporting patterns and safety signals of ASMs-related sleep disturbances using data from the FDA Adverse Event Reporting System (FAERS).

**Methods:**

We conducted a retrospective pharmacovigilance study using FAERS data from Q1 2004 to Q2 2025. Disproportionality analyses were performed to identify potential safety signals for sleep-related adverse events (sAEs) associated with ASMs. We further analyzed demographic characteristics, clinical manifestations, and time-to-onset profiles.

**Results:**

Analysis of 3,118 reports identified significant associations between multiple ASMs and sleep disturbances. Significant signals were detected for sodium channel blockers eslicarbazepine, stiripentol, and cenobamate, as well as for mechanistically diverse agents vigabatrin, pregabalin, brivaracetam, and cannabidiol. The study characterized a broad spectrum of over 30 distinct sleep disturbances, among which insomnia was the most frequently reported preferred term (n = 1,570).

**Conclusion:**

This pharmacovigilance study reveals significant associations between various ASMs and sleep disturbances. The distinct disproportionality reporting profiles identified for some agents, which differ from prior evidence, necessitate careful clinical interpretation. Overall, this study elucidates the complex sleep safety profiles of ASMs, offering evidence to support more informed drug selection and monitoring in practice.

## 1 Introduction

Sleep disturbances represent one of the most common comorbidities in patients with epilepsy (PWE) (Moore et al., 2021). According to available data, clinically significant sleep disturbances affect approximately one-third of epilepsy patients, corresponding to a prevalence rate approximately twice that found in the general population (Ismayilova et al., 2015). This elevated comorbidity arises from well-established bidirectional pathophysiological mechanisms: epileptic seizures disrupt sleep architecture and continuity (Grigg-Damberger and Foldvary-Schaefer, 2022), while sleep abnormalities in turn lower seizure thresholds and enhance neuronal hyperexcitability, establishing a self-perpetuating cycle of mutual exacerbation (Roliz and Kothare, 2023). Antiseizure medications (ASMs) form the therapeutic cornerstone for epilepsy, achieving adequate seizure control in approximately 70% of patients through optimized pharmacotherapy (Kanner and Bicchi, 2022). Recently, growing evidence has highlighted concerns regarding ASM-associated neuropsychiatric adverse effects, including mood disturbances, depression, anxiety, aggressive behavior, and suicidal ideation (De Bellis et al., 2025). Beyond these manifestations, the intricate interplay between epilepsy and sleep regulation has brought the impact of ASMs on sleep into focus as another critical consideration in treatment optimization.

Substantial evidence reveals remarkable heterogeneity in how different ASMs affect sleep parameters (Romigi et al., 2021; Carvalho et al., 2022). Certain medications demonstrate potentially beneficial effects on sleep quality that may complement their anticonvulsant efficacy, while others frequently precipitate adverse outcomes including sleep fragmentation, insomnia, or excessive daytime sedation (Liguori et al., 2021; Ye et al., 2022). Nevertheless, current understanding of ASM-specific sleep profiles remains substantially limited by inconsistent research findings and methodological constraints (Lawthom et al., 2023; Krutoshinskaya et al., 2024). Particularly concerning is the paucity of systematic safety data regarding sleep-related adverse events associated with newer-generation ASMs. Furthermore, existing evidence primarily derives from short-term observational studies with limited sample sizes, while randomized controlled trials typically employ restrictive inclusion criteria that systematically exclude elderly and multimorbid patients, who represent populations particularly vulnerable to medication-induced sleep disturbances (Romigi et al., 2021; Carvalho et al., 2022). These methodological limitations, combined with inadequate follow-up durations, substantially compromise the generalizability of existing evidence to real-world clinical practice.

The FDA Adverse Event Reporting System (FAERS) provides an unprecedented opportunity to address these evidence gaps through its comprehensive collection of spontaneously reported adverse drug events (Khaleel et al., 2022). This extensively validated pharmacovigilance database offers distinctive advantages for post-marketing safety surveillance, including massive sample sizes, population heterogeneity, and longitudinal data capture capabilities. Leveraging this robust repository, we conducted a systematic disproportionality analysis to elucidate associations between individual ASMs and specific sleep disturbances, aiming to generate reliable real-world evidence to guide personalized epilepsy management.

## 2 Methods

### 2.1 Data source

Data for this analysis were obtained from the FAERS database. The study interrogated reports for all ASMs with marketing approval in the United States, including eslicarbazepine, vigabatrin, oxcarbazepine, phenobarbital, phenytoin, perampanel, valproic acid, brivaracetam, cannabidiol, gabapentin, carbamazepine, lacosamide, lamotrigine, pregabalin, stiripentol, topiramate, cenobamate, levetiracetam, and zonisamide. Medication nomenclature (both generic and brand names) was validated against the FDA and DrugBank databases (https://go.drugbank.com/). The study period, from Q1 2004 to Q2 2025, was selected to encompass the complete post-approval timeline of all target ASMs, commencing with the earliest approved agent. Adverse events (AEs) were coded using the Medical Dictionary for Regulatory Activities (MedDRA, version 26.0, https://www.meddra.org/) and systematically classified according to System Organ Class (SOC), High Level Group Term (HLGT), High Level Term (HLT), and Preferred Term (PT). The specific Preferred Terms used to identify sleep-related adverse events are detailed in Supplementary Table S1.

### 2.2 Data processing procedure

**FIGURE 1:**
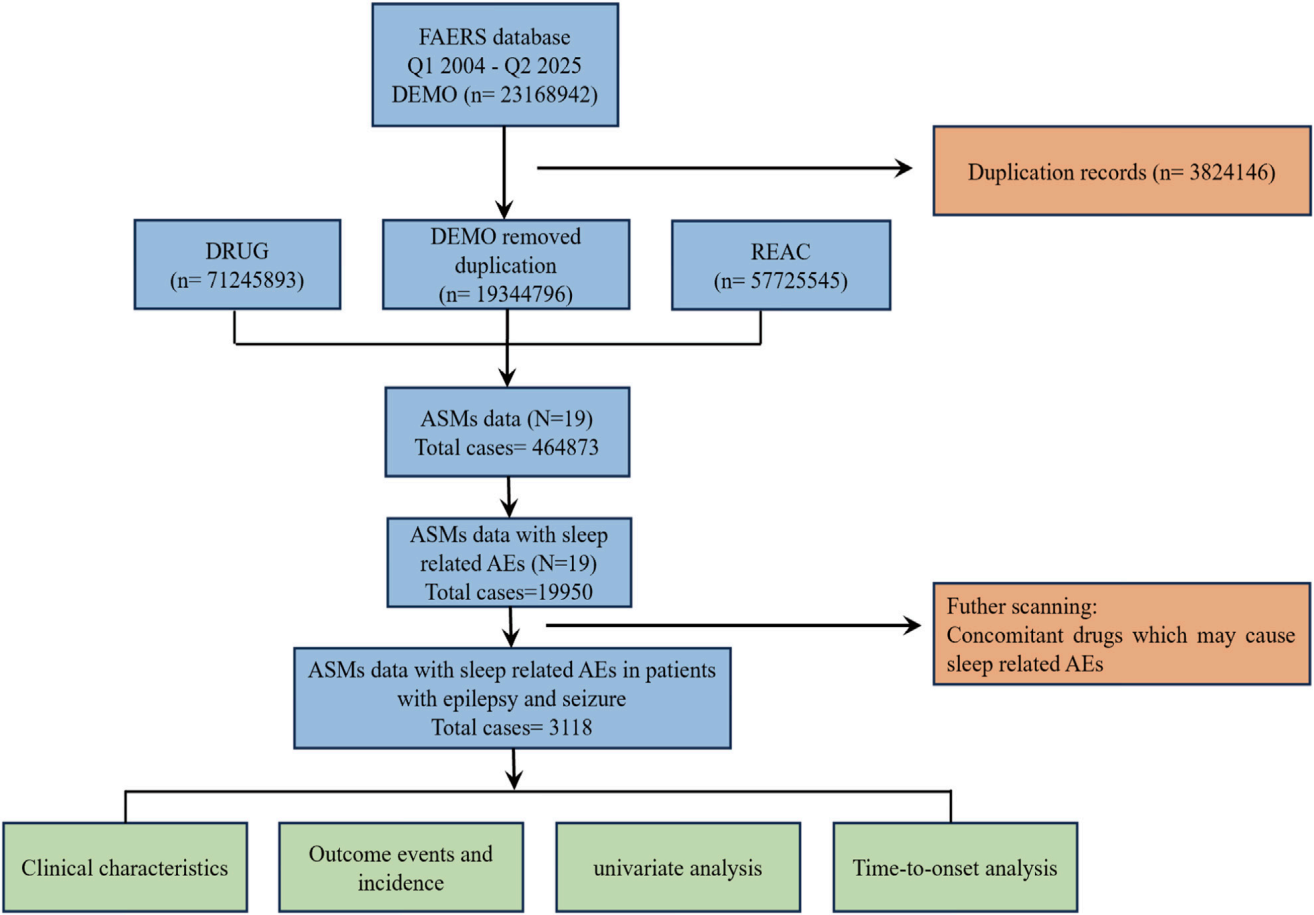
The flow chart illustrates the analytical procedure of the study. FAERS, FDA Adverse Event Reporting System; DEMO, demographic and administrative information; DRUG, drug information; REAC, adverse reaction information; ASMs, antiseizure medications; AEs, adverse events.

Raw ASCII data downloaded from the FAERS database were imported and processed to identify and remove duplicate reports according to the FDA-recommended deduplication strategy. A detailed flowchart is presented in Figure 1. Specifically, among reports sharing an identical CASEID, the entry with the most recent FDA_DT was retained; where both CASEID and FDA_DT matched, the record with the highest PRIMARYID was preserved. To improve signal specificity and exclude reports of sleep-related adverse events (sAEs) that might be caused by other factors, we restricted our analysis to reports where an ASM was the “Primary Suspect” and excluded a wide range of Concomitant medications known to cause sleep disturbances, including antipsychotics, opioids, muscle relaxants, antihypertensives, lipid-lowering agents, and non-steroidal anti-inflammatory drugs (NSAIDs), among others. The primary rationale for excluding each drug was based on direct evidence from the FDA prescribing information and support from the scientific literature (Cohrs, 2008; Valencia Carlo et al., 2023; Wang and Teichtahl, 2007; Dimsdale et al., 2007; Murphy et al., 1994; Monti, 1987; Szmyd et al., 2021; Wierzbiński et al., 2023; Ogeil and Phillips, 2015). A complete list of all excluded concomitant medications is provided in Supplementary Table S2. While this strategy reduces confounding from non-ASM factors, it may introduce selection bias, thereby limiting generalizability to patients with complex medication regimens. Furthermore, residual confounding may remain unaddressed. To ensure a systematic and comprehensive definition of relevant indications, we leveraged the MedDRA hierarchy. Our approach included all PTs classified under the HLGTs for “Epilepsy and seizure disorders nec,” “Partial simple seizures nec,” “Partial complex seizures,” “Absence seizures,” and “Generalised tonic-clonic seizures” within the “Nervous system disorders” SOC. This captured a broad spectrum of conditions, such as epilepsy, status epilepticus, Dravet syndrome, Lennox-Gastaut syndrome, atonic seizures, and generalized tonic-clonic seizures, among others. Only reports with indications listed among these pre-specified PTs were included in the analysis. Following deduplication, ASM-specific filtering, and indication-based inclusion, a final cohort of 3,118 unique AE reports associated with ASM therapy was derived for subsequent analysis.

### 2.3 Signal mining

The reporting odds ratio (ROR) was utilized to screen for potential associations between the target ASMs and specific AEs. The ROR quantifies the disproportionality in reporting frequency by comparing the proportion of a specific AE for a given drug against the background reporting proportion for that AE across all other drugs in the database (Rothman et al., 2004). In our analysis, to evaluate the associations between the target ASMs and sAEs, we defined the comparator group as all drugs reported in the FAERS database from Q1 2004 to Q2 2025 that were associated with sAEs, excluding the target ASMs. This approach aligns with standard practice in pharmacovigilance disproportionality analysis and aims to provide a broad and representative reference benchmark. The analysis is based on a 2 × 2 contingency table structure, which stratifies the database entries based on exposure to a particular drug (Drug X) and the occurrence of a specific AE (Event A) (Table 1). An elevated ROR suggests a stronger statistical association, indicating a potential safety signal. A signal was flagged as positive only if it satisfied both of the following pre-specified criteria: (1) at least three reported cases of the AE of interest; (2) a lower bound of the 95% confidence interval (CI) for the ROR >1. This dual-threshold approach improves the robustness of signal detection by minimizing spurious associations arising from random variability.

**TABLE 1:** Two-by-two contingency table for disproportionality analysis.

Item	Target AEs reported	Other AEs reported	Total
Target drugs	a	b	a + b
Other drugs	c	d	c + d
Total	a + c	b + d	a + b + c + d

AEs, adverse events; a, the number of case reports associating the target drug with the target AEs; b, the number of case reports involving the target drug but with all other AEs; c, the number of case reports associating all other drugs with the target AEs; d, the number of case reports involving all other drugs and all other AEs.

### 2.4 Statistical analysis

In the descriptive statistical analysis, categorical variables were presented as frequencies and proportions, while continuous variables were summarized as medians with interquartile ranges or means with standard deviations, based on their distribution characteristics. Factors such as age and gender, as well as the use of individual ASMs, were assessed for their association with ASMs-related sAEs. Univariate logistic regression analyses were performed for each factor to calculate the crude odds ratios (ORs). It is emphasized that these calculated ORs reflect reporting associations rather than measures of risk. Additionally, Kaplan-Meier methodology was used to plot the cumulative incidence curves for ASM-related sleep disturbances. A two-sided *p* < 0.05 was set as the threshold for statistical significance. All statistical analyses were performed using R software (version 4.2.3) within the RStudio environment.

## 3 Results

### 3.1 Descriptive analysis

We conducted a descriptive analysis of the baseline characteristics of 3,118 patients who experienced sAEs (Table 2). Demographically, the proportion of female patients (1,404 cases, 45.0%) was higher than that of male patients (1,064 cases, 34.1%). The majority fell within the 50–100 kg range (693 cases, 22.2%). The 18–64.9-year age group constituted the largest cohort (1,050 cases, 33.7%). In terms of clinical outcomes, the majority of AEs were categorized as “Other” (2,214 cases, 71.0%). Hospitalization was most common (698 cases, 22.4%) among serious outcomes. Reports originated primarily from consumers (1,677 cases, 53.8%). A marked increase in reporting was observed after 2016, with 75.8% of all cases reported between Q1 2016 and Q2 2025. Geographically, case distribution was highly skewed, with the United States contributing the majority of reports (1,743 cases, 55.9%).

**TABLE 2:** Clinical characteristics of sleep related AEs with ASMs in patients with epilepsy from the FAERS database (Q1 2004 - Q2 2025).

Characteristics	Patients with sAEs (N = 3118)	
Gender	Cases number	Percentage
Female	1404	45.00%
Male	1064	34.10%
Unknown	650	20.80%
Weight		
<50 kg	215	6.90%
50–100 kg	693	22.20%
>100 kg	89	2.90%
Unknown	2121	68.00%
Age		
<18	402	12.90%
18–64.9	1050	33.70%
65–85	275	8.80%
>85	40	1.30%
Unknown	1351	43.30%
Outcome		
Congenital anomaly	5	0.20%
Death	26	0.80%
Disability	72	2.30%
Hospitalization	698	22.40%
Life-threatening	103	3.30%
Other	2214	71.00%
Reporter type		
Consumer	1677	53.80%
Health professional	212	6.80%
Pharmacist	126	4.00%
Physician	687	22.00%
Unknown	416	13.30%
Year		
2004 Q1 -2009 Q4	208	6.70%
2010 Q1-2015 Q4	357	11.50%
2016 Q1-2020 Q4	1185	38.00%
2021 Q1-2025 Q2	1181	37.80%
Country (top 3)		
The United States	1743	55.90%
The United Kingdom	238	7.70%
Italy	148	4.70%

sAEs, sleep related adverse events; ASMs, antiseizure medications.

### 3.2 Disproportionality analysis

**FIGURE 2:**
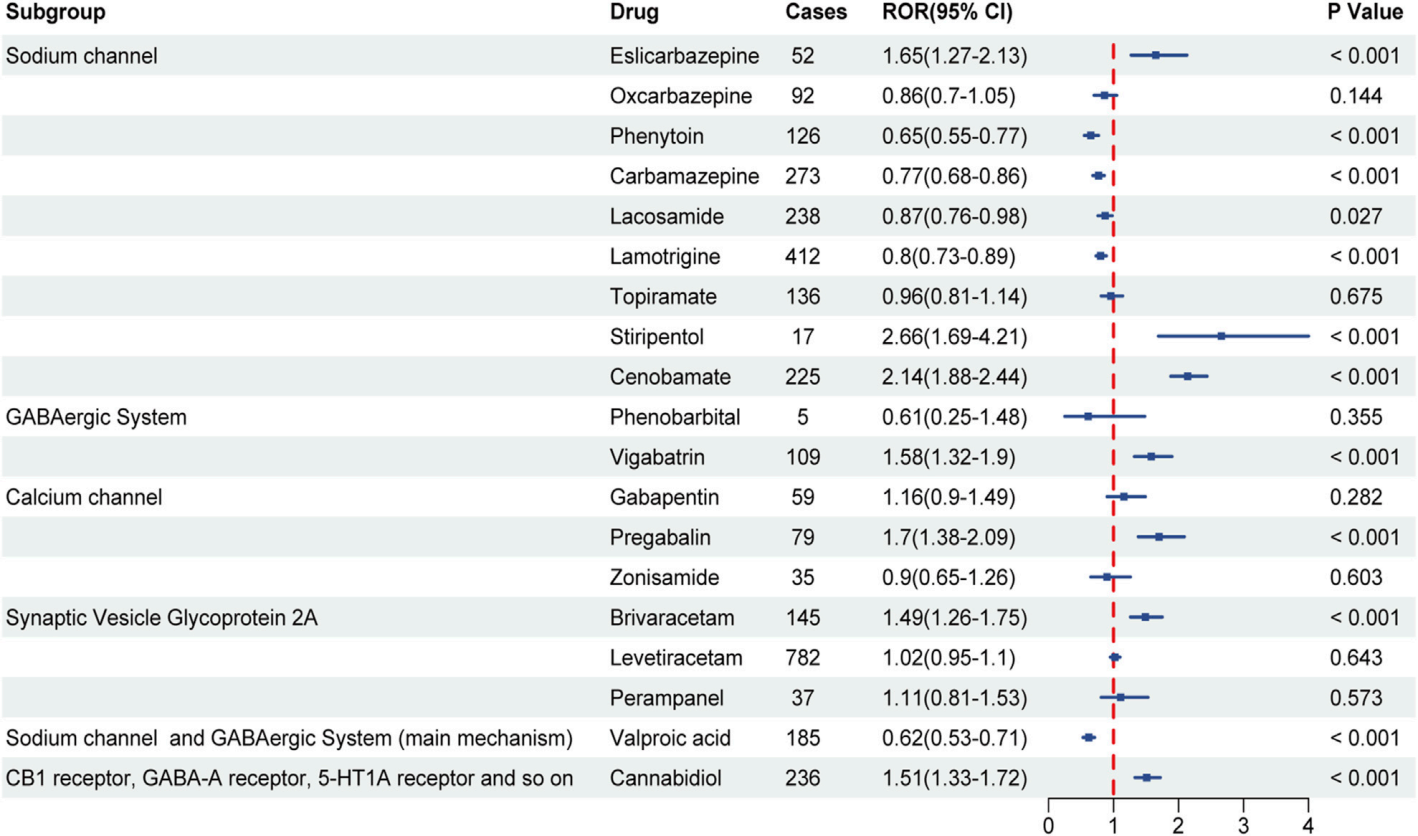
Disproportionality analysis of sleep-related adverse events (sAEs) associated with antiseizure medications (ASMs). Forest plot of reporting odds ratios (RORs) for sAEs associated with ASMs in the FAERS. Medications are stratified by their primary mechanism of action. The point estimate (ROR) for each drug is represented by a square, and the horizontal lines depict the 95% confidence intervals (CI).

Disproportionality analysis was performed to assess the associations between ASMs and sAEs. Substantial heterogeneity in RORs was observed across mechanistically distinct drug classes. Among sodium channel blockers, eslicarbazepine (ROR = 1.65, 95% CI 1.27–2.13), stiripentol (ROR = 2.66, 95% CI 1.69–4.21), and cenobamate (ROR = 2.14, 95% CI 1.88–2.44) exhibited statistically significant associations with sAEs, whereas phenytoin, carbamazepine, lacosamide, and lamotrigine showed no meaningful association (all RORs <1). Among agents with other mechanisms, several agents, including vigabatrin (ROR = 1.58, 95% CI 1.32–1.90), pregabalin (ROR = 1.70, 95% CI 1.38–2.09), brivaracetam (ROR = 1.49, 95% CI 1.26–1.75), and cannabidiol (ROR = 1.51, 95% CI 1.33–1.72) were also significantly associated with sAEs. In contrast, valproic acid was not associated with sAEs (ROR = 0.62, 95% CI 0.53–0.71). No statistically significant associations were detected for oxcarbazepine, topiramate, gabapentin, levetiracetam, or several other ASMs (Figure 2).

**FIGURE 3:**
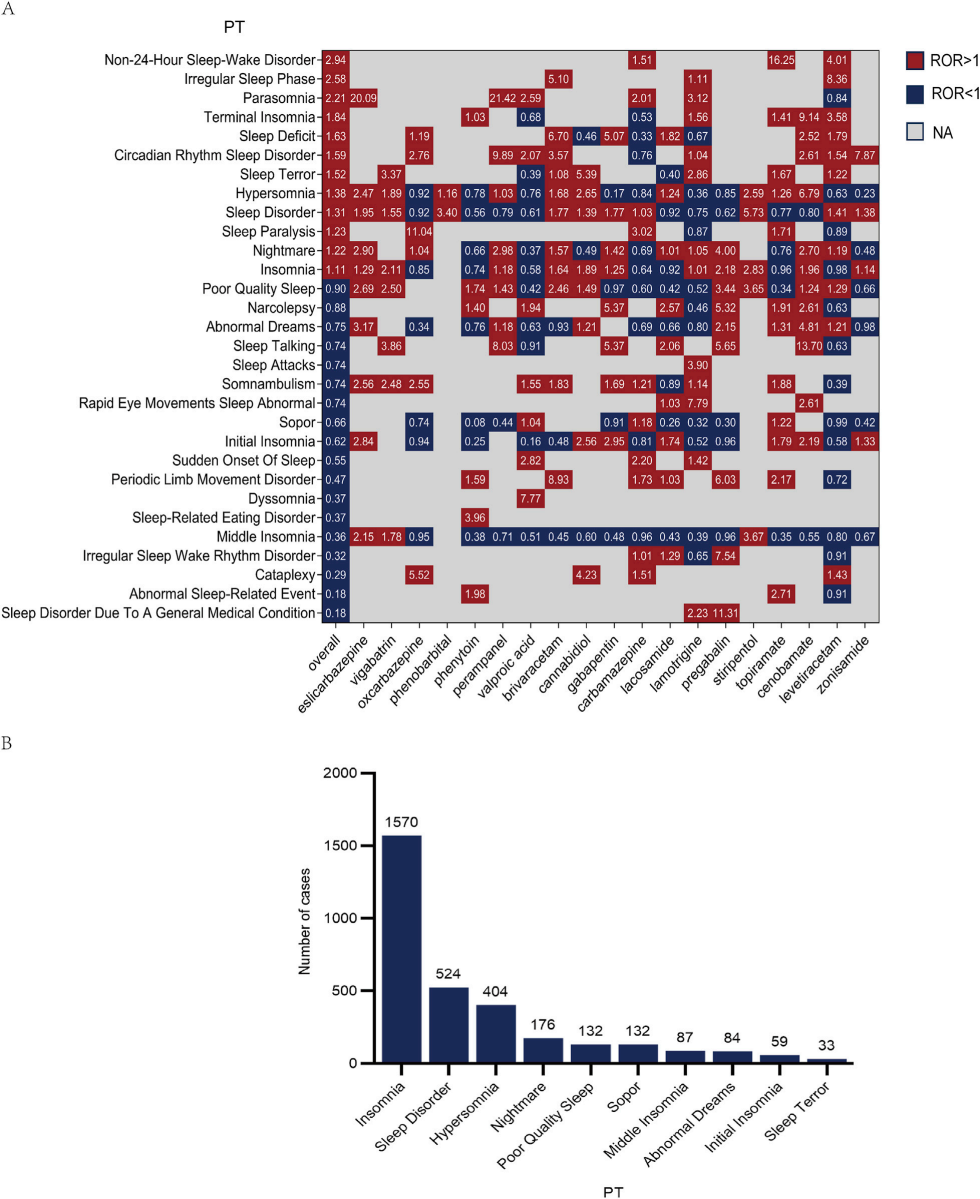
Signal detection of sleep-related adverse events (sAEs) for antiseizure medications (ASMs). **(A)** Heatmap visualizing the strength of association between individual ASMs and specific sAEs (Preferred Terms, PTs). With red hues representing positive signals (ROR >1) and blue hues representing negative signals (ROR <1), NA indicate missing data. The numerical value within each cell denotes the precise ROR **(B)** Bar chart showing the total number of reported cases for the top 10 most frequently reported sleep-related PTs across all studied ASMs.

We further characterized the spectrum of sAEs associated with ASMs (Figure 3A). Our analysis identified more than 30 specific PTs. Among these, insomnia was the most frequently reported event (1,570 cases) (Figure 3B). Drug-event association analysis revealed several highly significant disproportionality signals (ROR >10), including eslicarbazepine with parasomnia (ROR = 20.9, 95% CI 2.42–166.93), oxcarbazepine with sleep paralysis (ROR = 11.04, 95% CI 3.69–33.04), perampanel with parasomnia (ROR = 21.42, 95% CI 2.58–178.01), topiramate with non-24-h sleep-wake disorder (ROR = 16.25, 95% CI 4.06–64.99), cenobamate with sleep talking (ROR = 13.7, 95% CI 3.07–61.24), and levetiracetam with increased need for sleep (ROR = 20.06, 95% CI 2.24–179.52). However, the prominent signals described above were derived from a low number of case reports (a ≤4), necessitating further validation in subsequent studies. Comprehensive results are available in Supplementary Table S3.

**FIGURE 4:**
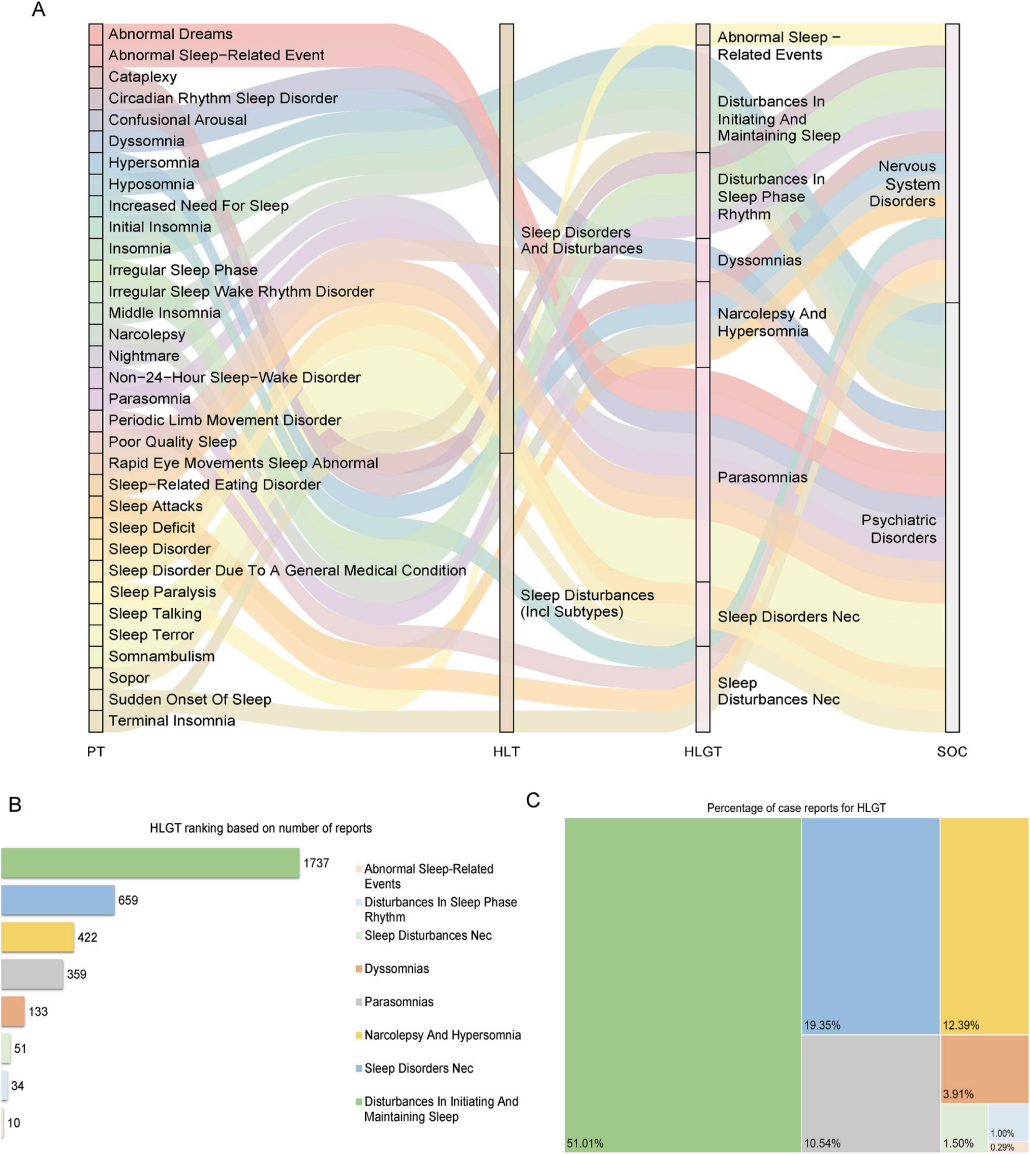
Hierarchical signal detection of sleep-related adverse events (sAEs) associated with antiseizure medications (ASMs). **(A)** Mulberry plot illustrating the hierarchical relationships of sleep-related Preferred Terms (PTs) within the MedDRA structure, connecting specific PTs to their respective High-Level Terms (HLTs) and High-Level Group Terms (HLGTs). **(B)** Bar chart ranking the top HLGTs by the absolute number of case reports, identifying the most frequently reported categories. **(C)** Treemap visualizing the proportional distribution of reports for each HLGT.

Figure 4A illustrates the hierarchical relationships among PTs, HLTs, HLGTs, and SOCs. Analysis at the HLGT level based on case reports identified “Disturbances in Initiating and Maintaining Sleep” as the most frequently reported category of sAEs, with 1737 cases (51.01%), followed by “Sleep Disorders NEC” (659 cases, 19.35%) and “Narcolepsy and Hypersomnia” (422 cases, 12.39%). Other notable categories included “Parasomnias” (359 cases, 10.54%), “Dyssomnias” (133 cases, 3.91%), “Sleep Disturbances Nec” (51 cases, 1.50%), “Disturbances in Sleep Phase Rhythm” (34 cases, 1.00%), and “Abnormal Sleep-Related Events” (10 cases, 0.29%) (Figures 4B,C).

### 3.3 Univariate logistic regression analysis

**FIGURE 5:**
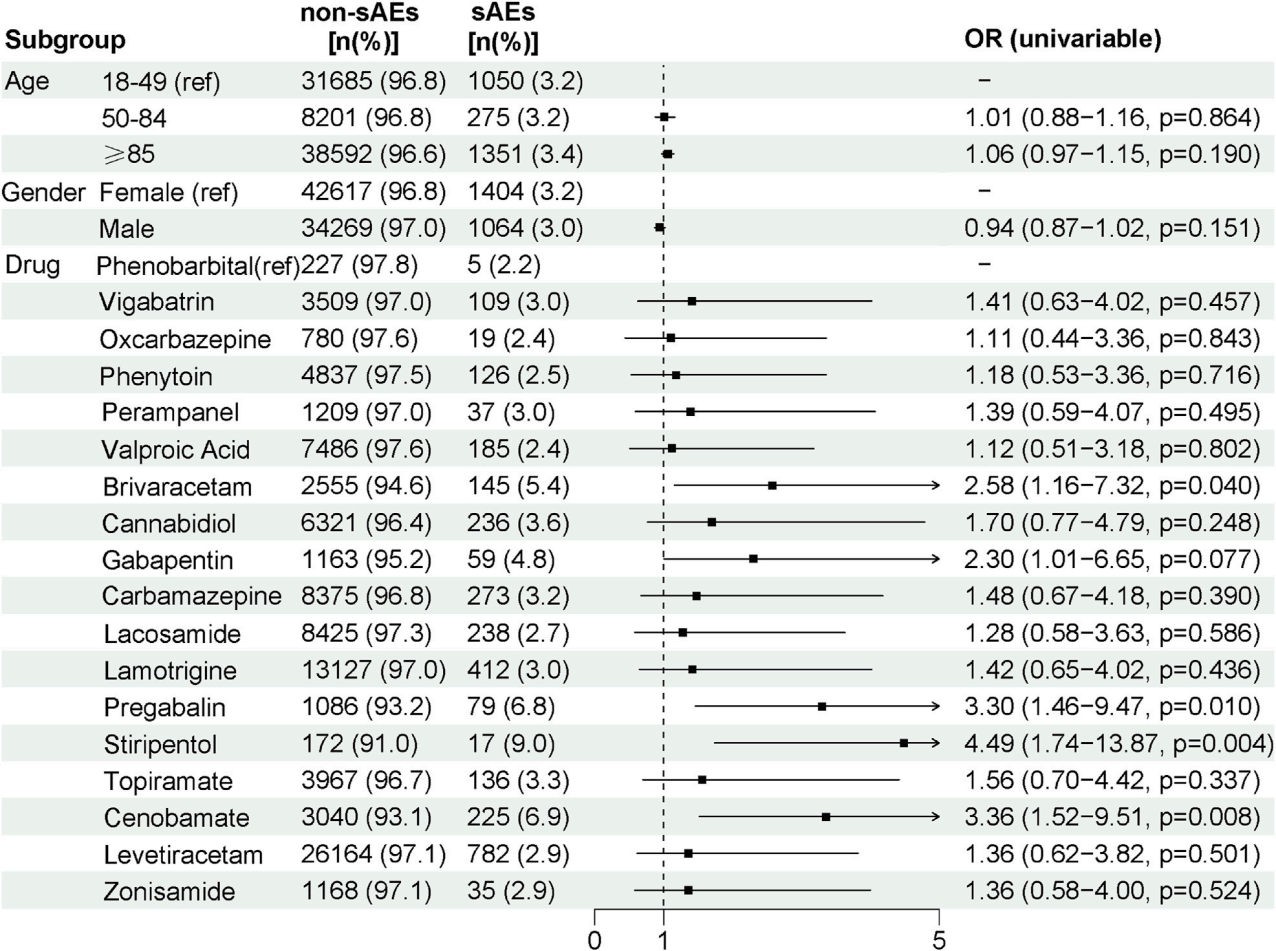
Univariable logistic regression analysis for ASMs related sAEs. This forest plot presents the results of a univariable logistic regression analysis identifying association of age, gender and individual ASMs with sAEs. The reference groups for each variable category are as follows: Age (18–49 years), Gender (Male), and Drug (Phenobarbital). The plot displays the odds ratio (OR) with 95% confidence interval for each variable.

We further performed univariable logistic regression to analyze the associations of age, sex, and individual ASMs with sAEs (Figure 5). The results indicated no significant associations between demographic factors and sAE reports: compared to females, males showed no significant difference in the odds ratio (OR) of sAEs; nor were significant differences observed in the odds of sAE across age groups (with 18–49 years as the reference). Among the ASMs analyzed against phenobarbital as the reference, several drugs showed significantly higher odds: stiripentol demonstrated the strongest association (OR = 4.49, 95% CI 1.74–13.87, *p* = 0.004), followed by cenobamate (OR = 3.36, 95% CI 1.52–9.51, *p* = 0.008), pregabalin (OR = 3.30, 95% CI 1.46–9.47, *p* = 0.010), and brivaracetam (OR = 2.58, 95% CI 1.16–7.32, *p* = 0.040). The remaining drugs showed a trend toward increased odds, though no statistically significant differences were detected.

### 3.4 Time to onset analysis of sAEs

**FIGURE 6:**
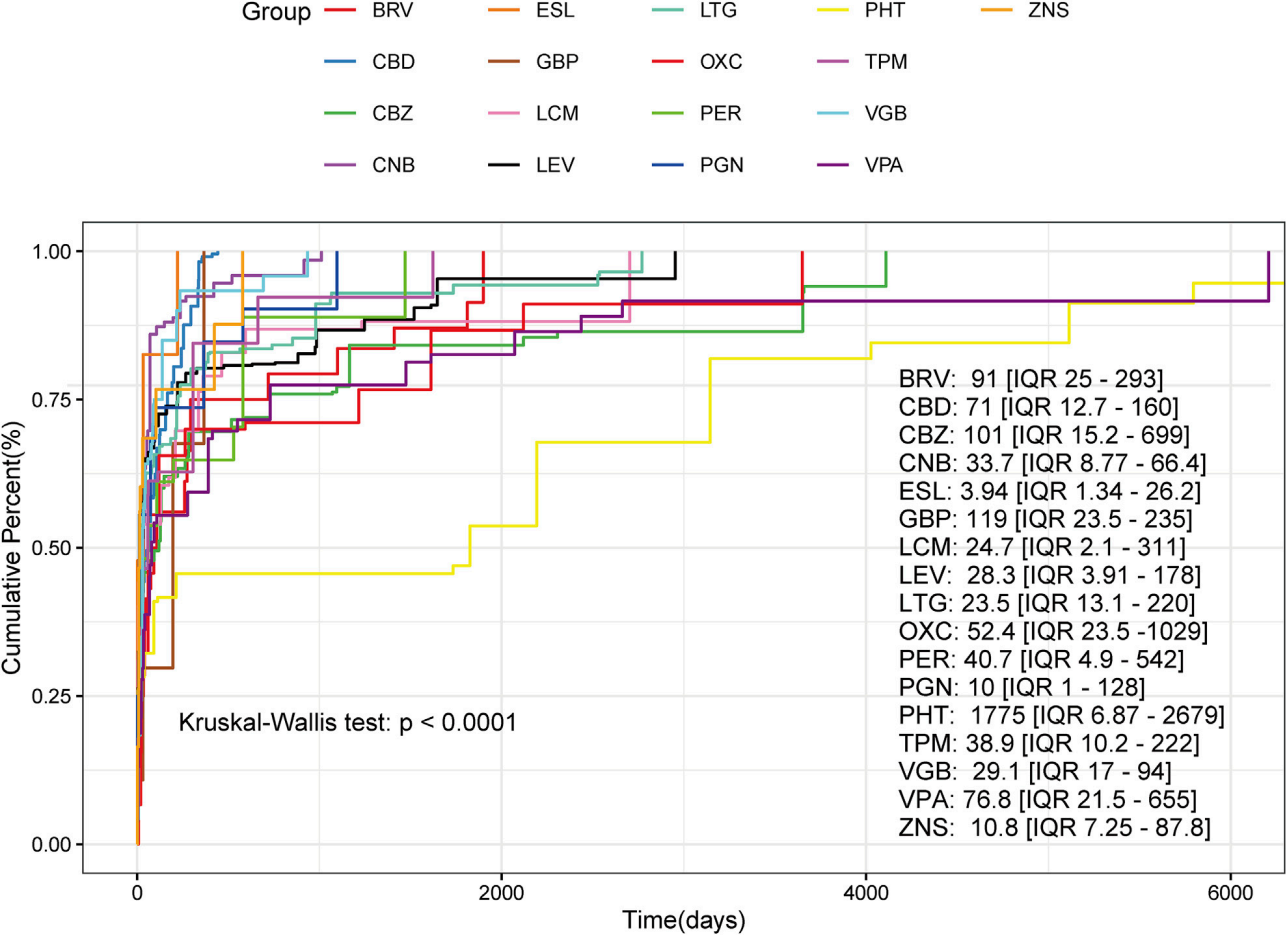
Time-to-onset profiles of sleep-related adverse events (sAEs) for antiseizure medications (ASMs). Kaplan–Meier cumulative incidence curves illustrate the onset time of sAEs across 17 different ASMs. Each curve represents the cumulative probability of sAE occurrence over time for a specific drug, with the median time-to-onset and interquartile range (IQR) annotated for each. The Kruskal–Wallis test revealed statistically significant differences in time-to-onset distributions among all drugs (p < 0.0001). ESL: eslicarbazepine; VGB: vigabatrin; OXC: oxcarbazepine; PB: phenobarbital; PHT: phenytoin; PER: perampanel; VPA: valproic acid; BRV: brivaracetam; CBD: cannabidiol; GBP: gabapentin; CBZ: carbamazepine; LCM: lacosamide; LTG: lamotrigine; PGN: pregabalin; TPM: topiramate; LEV: levetiracetam; ZNS: zonisamide; CNB: cenobamate.

We compared the time to sAEs among patients receiving different ASMs. Survival curves diverged significantly across the 17 drugs (Kruskal–Wallis test, *p* < 0.0001), indicating that the choice of ASM strongly influenced sAE timing (In the analysis of stiripentol and phenobarbital, a time-to-onset curve could not be generated due to a lack of data). The median time to sAE onset varied substantially, ranging from 3.94 days (IQR 1.34–26.2) for eslicarbazepine to 1775 days (IQR 6.87–2679) for phenytoin. Pregabalin (10 days) and zonisamide (10.8 days) also demonstrated notably shorter time to sAE occurrence compared with other agents. However, it is important to note that the analysis was necessarily limited to cases with documented onset dates, which represented a variable proportion of total reports for each drug (ranging from 6.8% to 32.4%) (Supplementary Table S4). Given concerns regarding data completeness, the interpretation of these temporal differences across medications should be approached with caution. (Figure 6).

## 4 Discussion

While treatment-emergent sleep disturbances associated with ASMs have been occasionally reported, a comprehensive, large-scale characterization of these events across different ASMs has been lacking. Our study addressed this gap by conducting a systematic, real-world pharmacovigilance investigation of the FAERS database. We provided a class- and drug-specific profile of sleep disturbances in PWE, identifying distinct reporting signals and substantial heterogeneity in clinical manifestations across 19 commonly used ASMs. This analysis offers a foundational framework for understanding the spectrum of ASM-associated sleep disturbances in clinical practice.

This study systematically evaluated major categories of ASMs, including sodium channel modulators, GABAergic system modulators, calcium channel modulators, SV2A modulators, and multi-target agents. Notably, the analysis revealed that even drugs with similar or identical mechanisms of action exhibited distinct associations with sleep disturbances, underscoring the importance of drug-specific evaluation. Among sodium channel blockers, carbamazepine, lacosamide, and lamotrigine generally demonstrated no significant signals of disproportionate reporting for sleep disturbances, consistent with previous literature. Specifically, the impact of carbamazepine on sleep structure was primarily characterized by transient AEs during initial treatment, with longitudinal studies confirming that both sleep macrostructure and daytime alertness returned to baseline levels within 1 month of treatment (Nayak et al., 2016; Jain and Glauser, 2014). Furthermore, clinical studies consistently demonstrated excellent sleep safety profiles for lacosamide. In randomized controlled trials involving both healthy subjects and adults with focal epilepsy, lacosamide showed no clinically significant AEs on polysomnography-measured sleep architecture, subjective daytime sleepiness, or sleep quality (Foldvary-Schaefer et al., 2017). Importantly, some parameters even showed stability or modest improvement, confirming its ability to maintain sleep-wake cycle integrity while controlling seizures (Foldvary-Schaefer et al., 2017; Lupo et al., 2023). Additionally, lamotrigine exhibited predominantly positive effects on sleep architecture in PWE (Legros and Bazil, 2003). Polysomnographic studies clearly demonstrated that adjunctive therapy significantly prolonged rapid eye movement (REM) sleep duration while reducing sleep stage transitions and arousal indices, thereby effectively enhancing sleep continuity and stability. Crucially, these improvements were not accompanied by significant increases in daytime sleepiness (Bazil, 2017; Kataria and Vaughn, 2016; Foldvary et al., 2001; Jain and Glauser, 2014). Overall, lamotrigine demonstrates favorable sleep safety characteristics by improving nocturnal sleep architecture while maintaining daytime alertness levels.

In contrast, our analysis indicates that phenytoin use shows no significant association with sleep disturbances. However, available evidence reveals that impact of phenytoin on sleep architecture demonstrates complexity and multidimensional characteristics. Specifically, some studies report reduced N1 sleep and enhanced slow-wave sleep (Staniszewska et al., 2017), while others observe increased N1 sleep along with suppression of both slow-wave and REM sleep (Lanigar and Bandyopadhyay, 2017). Similarly, we also observed divergent results with phenobarbital (Iivanainen and Savolainen, 1983; Wolf et al., 1984; Liguori et al., 2021). These discrepancies may be closely related to differences in study design, population characteristics, and pharmacokinetic parameters, reflecting the complexity of the neurophysiological effects for drugs and interindividual variability. Meanwhile, the analysis results for oxcarbazepine and topiramate warrant attention. Neither drug showed a significant reporting of sleep disturbances in our findings. However, detailed literature analysis reveals that oxcarbazepine exhibits definite sedative properties, characterized by shortened sleep latency, increased total sleep time and REM sleep, along with higher rates of reported daytime somnolence (Ayala-Guerrero et al., 2009; Thelengana et al., 2019). In contrast, although current research on sleep effects of topiramate remains relatively limited, available evidence suggests that for newly diagnosed epilepsy patients, low-dose, slowly titrated topiramate monotherapy can effectively control seizures without significantly affecting daytime alertness, demonstrating good clinical tolerability (Bonanni et al., 2004). Particularly noteworthy was our finding that eslicarbazepine showed a signal of disproportionate reporting of sleep disturbances, which differs from the conclusion reached by Romigi et al. (2020) based on polysomnographic improvements in sleep microstructure. This discrepancy may stem from differences in assessment dimensions (subjective experience versus objective parameters), study population characteristics, and medication background factors. It should be clarified that our findings do not negate previous conclusions but rather suggest the drug may have dual effects on sleep: potentially improving sleep stability while possibly causing subjective sleep quality deterioration in some patients. Therefore, this hypothesis requires further validation through future trials incorporating both subjective and objective measures in large sample studies. Regarding other ASM classes, our analysis clearly confirms particularly strong associations between sleep disturbances and both stiripentol and cenobamate. Specific data from pre-clinical trials of stiripentol show that 67% of the treatment group reported somnolence symptoms compared to only 23% in the placebo group, with insomnia rates of 12% and 7% respectively (US Food and Drug Administration, 2022). Similarly, cenobamate also causes definite somnolence adverse reactions, requiring particular attention and monitoring during clinical use (US Food and Drug Administration, 2025). The impact of valproic acid on sleep architecture remains controversial. Our results show no statistically significant association between valproate therapy and sleep disturbances. Research on valproic acid reveals a complex and contradictory profile of its effects on sleep architecture. In studies on juvenile myoclonic epilepsy (JME), valproic acid monotherapy has been associated with both beneficial changes, such as increased sleep efficiency, prolonged N2 and N3 sleep, and extended REM duration, and neutral outcomes, such as unchanged sleep latency or daytime sleepiness (Nayak et al., 2015). Conversely, a study in mixed epilepsy types reported consistently disruptive effects, including prolonged N1 sleep, shortened REM sleep, and decreased sleep efficiency, particularly with long-term use (>3 months) (Zhang et al., 2014). This paradox is further highlighted in childhood absence epilepsy (CAE), where sleep outcomes were directly tied to seizure control, improving in fully controlled patients but worsening in those with partial control (Glauser et al., 2013). These findings collectively suggest that the impact of valproic acid on sleep is not uniform but is significantly influenced by epilepsy type, treatment duration, and therapeutic response.

Both brivaracetam and levetiracetam target synaptic vesicle protein 2A, whereas perampanel is a selective AMPA receptor antagonist. Notably, our study found a significant association between brivaracetam and sleep disturbances based on disproportionality analysis, which was not observed for levetiracetam and perampanel. This finding is corroborated by evidence from randomized controlled trials of brivaracetam, in which approximately 20% of participants reported daytime somnolence (Klein et al., 2015). In contrast, some clinical studies have suggested potential sleep-improving effects (Klein and Bourikas, 2024; Faught et al., 2024). However, the methodological limitations and considerable sample heterogeneity in these investigations necessitate cautious interpretation of such results. Overall, the convergence between our real-world pharmacovigilance data and previous controlled trial evidence strengthens the validity of the observed safety signal for brivaracetam. Nevertheless, further studies employing standardized methodologies and larger cohorts remain essential to fully elucidate the safety profiles of these drugs on sleep. In contrast, levetiracetam demonstrates a clear dosedependent effect on sleep. Research indicates that a gradually titrated regimen reaching 2000 mg daily can significantly improve sleep quality, manifested as extended total sleep time, improved sleep efficiency, and enhanced deep sleep (Cicolin et al., 2006; Chaneva, 2021; Thelengana et al., 2019). Importantly, these improvements occur without compromising daytime alertness, suggesting good tolerability and potential sleep-enhancing properties. Meanwhile, perampanel shows positive effects in improving sleep for epilepsy patients. Studies confirm that adjunctive perampanel treatment not only objectively optimizes sleep architecture by increasing deep sleep proportion and improving sleep efficiency, but also significantly enhances subjective sleep quality and alleviates insomnia symptoms without causing daytime drowsiness (Rocamora et al., 2020; Bergamo et al., 2025). For gabapentin, or zonisamide, our study observed no significant associations with sleep disturbances, consistent with previous research findings (Romigi et al., 2021; Carvalho et al., 2022). However, vigabatrin and pregabalin demonstrated distinct risk signals for sleep disturbances in our analysis. This finding contrasts with some conventional perspectives suggesting that vigabatrin as adjunctive therapy to carbamazepine does not significantly exacerbate daytime sleepiness (Bonanni et al., 1997). It should be noted that studies supporting this conventional conclusion are often limited by small sample sizes, which may compromise their ability to detect potential risk signals. Therefore, further investigation with higher-level evidence and larger sample sizes is warranted to validate this association. Previous literature suggests that pregabalin may offer potential benefits for sleep in patients with epilepsy, primarily reflected in the improvement of sleep architecture and quality. Some studies indicate that it can enhance sleep depth by increasing slow-wave sleep (deep sleep) and reducing the proportion of light sleep, as well as consolidate sleep maintenance by decreasing the number of nocturnal awakenings (Romigi et al., 2021). Our study failed to replicate these findings. We cautiously speculate that the discrepancy between previous conclusions and our results may be attributable to methodological limitations, along with potential factors such as reporting bias (e.g., misreporting by participants) in the original studies. Cannabidiol exhibits distinct population-dependent effects on sleep. While the drug shows no significant disruption to physiological sleep in healthy populations (Linares et al., 2018), it demonstrates potential for improving sleep microstructure in children with drug-resistant epilepsy. Specifically, Klotz et al. (2021) reported that after 3 months of cannabidiol treatment, over 80% of children with initial abnormalities showed improvements in sleep spindle and slow-wave activity parameters. An open-label study by Anderson et al. (2021) further confirmed that 12 months of cannabidiol treatment significantly improved sleep duration, nocturnal arousals, and daytime sleepiness. However, our analysis identified a signal of disproportionate reporting of sleep disturbances associated with cannabidiol, which contradicts existing research findings. It should be noted that studies supporting the sleep-improving effects of brivaracetam are predominantly open-label and limited in sample size, potentially influenced by expectation bias and population heterogeneity. Therefore, the discrepancies in current conclusions may stem from multiple factors, including study populations, drug dosage, concomitant medications, and research design, necessitating further validation through more rigorous randomized double-blind controlled trials.

Through a comprehensive analysis of ASM-related sAEs, we documented over 30 different sleep-related symptoms, establishing a complete clinical phenotypic spectrum. At the HLGT level, sleep initiation and maintenance disorders were the most common category (51.01%). This distribution pattern is primarily attributed to the preferred term “insomnia” within its subordinate level, with this single symptom alone accounting for 50.4% (1570/3118) of the total reports. This finding aligns with the known properties of ASMs, which have broad neuromodulatory effects and may interfere with sleep-wake pathways (Wang et al., 2018). Notably, such as nightmares, sleep paralysis, and sleep terror, which, although reported relatively infrequently, may profoundly impact patients’ quality of life, mental health, and treatment adherence. Through disproportionality analysis, we detected several potential pharmacovigilance signals (ROR >10). It is crucial to emphasize that these findings represent statistical associations based on a spontaneous reporting system, and their clinical significance requires further validation through additional research. Among these signals, the association between perampanel and sleep disturbances was particularly notable (ROR = 21.42). A similar signal was observed for eslicarbazepine and sleep disturbances (ROR = 20.09). For these drugs, it is recommended that clinicians proactively inquire about changes in patients’ sleep quality and consider enhanced follow-up during the initial treatment phase. Several new findings with clear clinical implications warrant special attention. A significant association was observed between oxcarbazepine and sleep paralysis (ROR = 11.04), a transient state occurring during sleep-wake transitions characterized by voluntary muscle paralysis and vivid hallucinations such as sensed presence or chest pressure, suggesting potential interference with REM-wake transition mechanisms (Denis et al., 2018). Clinicians prescribing oxcarbazepine should proactively inquire about such symptoms, particularly during treatment initiation or dose adjustment, to facilitate early intervention and mitigate impacts on sleep quality and treatment adherence. Additionally, topiramate showed a notable association with Non-24-Hour Sleep-Wake Rhythm Disorder (ROR = 16.25), indicating possible circadian rhythm disruption via mechanisms like carbonic anhydrase inhibition (Dodgson et al., 2000; Parkkila et al., 1995). Patients on long-term topiramate therapy reporting alternating insomnia and excessive daytime sleepiness should be evaluated for circadian rhythm disturbances. Furthermore, adjusting the dosing schedule may serve as a management strategy for these individuals. Furthermore, levetiracetam demonstrated a strong association with increased sleep need (ROR = 20.06), underscoring the importance of pre-treatment counseling about daytime sleepiness, assessing its functional impact, and warning against high-alertness activities during therapy. These signals provide valuable references for clinical practice, but their causal relationships and clinical significance need further confirmation through prospective studies and larger samples. Future research should focus on elucidating the underlying mechanisms of these associations and providing clinicians with evidence-based management guidelines.

Time-to-event analysis revealed differences in the timing of drug-related signals; however, interpretation must account for potential issues such as recall bias, missing data, and left truncation. In this study, the proportion of cases with complete onset data ranged from 6.8% (gabapentin) to 32.4% (perampanel), with most drugs below 25%, indicating that incomplete data are common. Against this background, drugs such as eslicarbazepine (9.6%), pregabalin (10.1%), and zonisamide (25.7%) exhibited early disproportional reporting patterns, with a median time to onset of approximately 10 days, suggesting a possible association with rapid pharmacokinetic attainment or acute pharmacodynamic effects. In contrast, phenytoin (14.3%) demonstrated a markedly delayed reporting pattern, with a median onset time exceeding 4 years, which was significantly longer than the 1–3 months observed for most other drugs. It should be noted that date entries in spontaneous reporting systems are often incomplete or inconsistent. The exceptionally delayed onset observed with phenytoin may particularly reflect reporting artifacts, such as recall bias, rather than a true delayed pharmacodynamic effect. Despite these limitations, the observed temporal patterns still suggest the value of individualized monitoring strategies, such as intensified follow-up for early-signal drugs and long-term monitoring mechanisms for delayed-signal drugs; however, all such strategies must be developed with full awareness of the inherent uncertainties in spontaneous reporting data.

Several limitations of this study should be acknowledged. First, disproportionality analysis can only indicate statistical associations rather than establish causal relationships. Second, the FAERS database, as a spontaneous reporting system, is subject to several inherent biases. These include selective reporting bias, potential underreporting of certain events, overreporting influenced by media attention or regulatory actions, and channeling bias (where drugs are preferentially prescribed to patients with specific characteristics). Additionally, the absence of comprehensive denominator data precludes estimation of true incidence or prevalence rates, and thus findings should not be interpreted as reflecting actual population risks. Important clinical confounders such as disease severity, specific ASM dosage, treatment duration, and detailed patient comorbidities are not routinely available, which could influence the observed associations. Furthermore, the wide confidence intervals for rare events reflect inherent estimation uncertainty.

## 5 Conclusion

This study, through a large-scale real-world analysis, systematically elucidates the complex associations between ASMs and sleep disturbances. Our findings confirm that ASMs with distinct mechanisms of action exhibit differentiated sleep safety profiles. We characterized drug-specific disproportionality reporting signals and their temporal dynamics, established a clinical phenotype spectrum encompassing over 30 types of sleep disturbances, and identified multiple novel drug-event associations. It should be noted that these findings, derived from spontaneous reporting data, indicate statistical associations rather than established causal relationships and do not support direct estimation of population-level risks. Future work should prioritize validation of stratified monitoring strategies through prospective controlled studies and investigation of underlying mechanisms. The ultimate goal remains to balance seizure control with sleep safety, thereby improving the overall quality of life for people with epilepsy.

## Data Availability

The datasets presented in this study can be found in online repositories. The names of the repository/repositories and accession number(s) can be found in the article/Supplementary Material.
